# Mer590, a novel monoclonal antibody targeting MER receptor tyrosine kinase, decreases colony formation and increases chemosensitivity in non-small cell lung cancer

**DOI:** 10.18632/oncotarget.2142

**Published:** 2014-06-26

**Authors:** Christopher T. Cummings, Rachel M.A. Linger, Rebecca A. Cohen, Susan Sather, Gregory D. Kirkpatrick, Kurtis D. Davies, Deborah DeRyckere, H. Shelton Earp, Douglas K. Graham

**Affiliations:** ^1^ Department of Pediatrics, Section of Hematology, Oncology, and Bone Marrow Transplantation, University of Colorado Anschutz Medical Campus, Aurora, CO, USA; ^2^ Department of Medicine, UNC Lineberger Comprehensive Cancer Center, Chapel Hill, NC, USA; ^3^ Department of Pharmacology, School of Medicine, University of North Carolina at Chapel Hill, Chapel Hill, NC, USA; ^4^ Department of Biomedical Sciences, Rocky Vista University College of Osteopathic Medicine, Parker, CO, USA

**Keywords:** MER, NSCLC, Monoclonal Antibody, Chemosensitivity, Targeted Therapy

## Abstract

The successes of targeted therapeutics against EGFR and ALK in non-small cell lung cancer (NSCLC) have demonstrated the substantial survival gains made possible by precision therapy. However, the majority of patients do not have tumors with genetic alterations responsive to these therapies, and therefore identification of new targets is needed. Our laboratory previously identified MER receptor tyrosine kinase as one such potential target. We now report our findings targeting MER with a clinically translatable agent – Mer590, a monoclonal antibody specific for MER. Mer590 rapidly and robustly reduced surface and total MER levels in multiple cell lines. Treatment reduced surface MER levels by 87%, and this effect was maximal within four hours. Total MER levels were also dramatically reduced, and this persisted for at least seven days. Mechanistically, MER down-regulation was mediated by receptor internalization and degradation, leading to inhibition of downstream signaling through STAT6, AKT, and ERK1/2. Functionally, this resulted in increased apoptosis, increased chemosensitivity to carboplatin, and decreased colony formation. In addition to carboplatin, Mer590 interacted cooperatively with shRNA-mediated MER inhibition to augment apoptosis. These data demonstrate that MER inhibition can be achieved with a monoclonal antibody in NSCLC. Optimization toward a clinically available anti-MER antibody is warranted.

## INTRODUCTION

Optimization of conventional chemotherapy regimens has led to modest gains in survival in NSCLC over the past few decades, and studies suggest that new treatment strategies must be pursued in order to achieve more impressive clinical gains [1-3]. Toward this end, therapies that target specific molecular aberrations in NSCLC cells have begun to emerge. Small molecule tyrosine kinase inhibitors directed against mutated EGFR or ALK fusion proteins have transformed treatment outcomes for the ~10% and ~4%, respectively, of Western NSCLC patients whose tumors are driven by one of these activated oncogenes. This has improved survival over conventional chemotherapeutics while offering a more tolerable side effect profile [4-8]. Monoclonal antibodies are another method to specifically target proteins that promote tumorigenesis or allow tumor survival. Thirteen antibodies are currently FDA approved for the treatment of various neoplastic diseases, with many more in various stages of pre-clinical development [9]. Only one, the anti-VEGF-A antibody bevacizumab, is FDA approved for the treatment of NSCLC, in combination with paclitaxel and carboplatin [10]. Despite these advances, targeted therapies are not currently available for the majority of patients, whose tumors are not driven by these specific molecular aberrations. Identification of novel targets is therefore a priority in order to increase the number of patients who will benefit from biologically-oriented therapeutics.

One family of proteins that has gained increased attention as a possible target for cancer therapy is the TAM family, composed of the receptor tyrosine kinases TYRO3, AXL, and MER. Monoclonal antibodies against TYRO3 have demonstrated efficacy in pre-clinical melanoma models, and antibodies against AXL have been efficacious in breast and lung cancer models [11,12]. The third member of the family, MER, has also been validated as a potential drug target in cancer. MER is over-expressed or aberrantly expressed in a wide variety of human malignancies [13-21]. Importantly, MER activates an extensive network of pro-oncogenic downstream signaling pathways mediating survival, proliferation, and migration of cancer cells [22]. In NSCLC in particular, MER is over-expressed in approximately two-thirds of patient tumors, regardless of histology, and inhibition of MER by shRNA in NSCLC cell lines promoted apoptosis and reduced colony formation in soft agar, as well as prevented *in vivo* tumor growth in a murine subcutaneous xenograft model [13]. These data suggest that development of clinically relevant MER inhibitors is warranted. Our group has been developing MER-selective small molecule inhibitors, and in this study we report our efforts to target MER using a novel monoclonal antibody, Mer590 [19, 23-26]. We have previously demonstrated that Mer590 decreases glioblastoma cell migration *in vitro* [27]. Here we further advance the case for development by providing pre-clinical evidence characterizing its mechanism of action, its effects on downstream signaling, and its combinatorial effects with conventional chemotherapy and a second mechanism of MER inhibition in NSCLC cells.

## RESULTS

### Mer590 Decreases Total Cellular and Surface MER Expression

**Figure 1:**
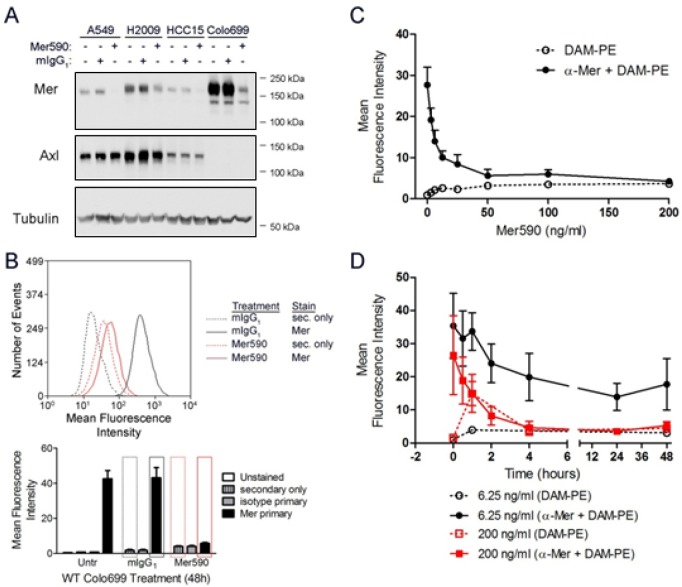
A novel inhibitory anti-MER antibody, Mer590, reduces total cellular and surface expression of MER (A) A549, H2009, HCC15, and Colo699 cells were cultured in the presence of 0.5 μg/ml Mer590, 0.5 μg/ml isotype control murine immunoglobulin (mIgG_1_), or PBS vehicle control for 24 hours. Western blot analysis demonstrated significant loss of MER protein expression after Mer590 treatment without affecting expression of the related receptor tyrosine kinase AXL. Tubulin was used as a loading control. (B) Colo699 cultures were treated with 0.5 μg/ml Mer590 or mIgG_1_ for 48 hours, and then stained for surface MER expression and analyzed by flow cytometry. Representative histograms (top panel) correspond to red and black rectangles overlaying the bar graph (bottom panel). (C) Colo699 cultures were treated for 48 hours with the indicated doses of Mer590 or with vehicle control and surface MER protein was detected by flow cytometry. (D) Colo699 cells were treated with 6.25 ng/ml Mer590, 200 ng/ml Mer590, or vehicle control for the indicated times and surface MER levels were determined by flow cytometry. Mean values and standard errors were derived from 3 independent experiments.

We generated a novel monoclonal antibody, Mer590, against the extracellular domain of human MER in mouse hybridoma cells [27]. A 24-hour exposure to 0.5 μg/ml Mer590 significantly reduced MER total protein levels in four NSCLC cell lines, without affecting levels of the closely related receptor tyrosine kinase, AXL (Figure 1A). Comparable results were obtained after 48 hours of Mer590 treatment (data not shown). Additional experiments with HCC15 cells demonstrated persistent knockdown of MER seven days after a single application of Mer590 (data not shown). As total MER decrease was consistent in all four NSCLC cell lines assayed, we selected two representative cell lines for further study: Colo699 because it does not express AXL and is MER-dependent, and H2009 as a representative cell line expressing both MER and AXL. Like total MER expression, surface MER expression as measured by flow cytometry was also decreased after Mer590 treatment, with a reduction of 87% after 48 hours of treatment in the Colo699 cell line (Figure 1B). Dose-response curves were generated, and indicate that a concentration of 6.25 ng/ml of Mer590 was sufficient to decrease MER surface levels by 50%, while a concentration of 50 ng/ml was sufficient to produce the maximal decrease in MER surface levels (Figure 1C). Finally, the kinetics of the Mer590-induced decrease in MER surface levels were determined, again by surface flow cytometry. At concentrations of 6.25 or 200 ng/ml of Mer590, maximal reduction of surface MER was achieved within four hours of Mer590 exposure, independent of the dose (Figure 1D).

### Mer590 Induces Receptor Internalization of MER

**Figure 2:**
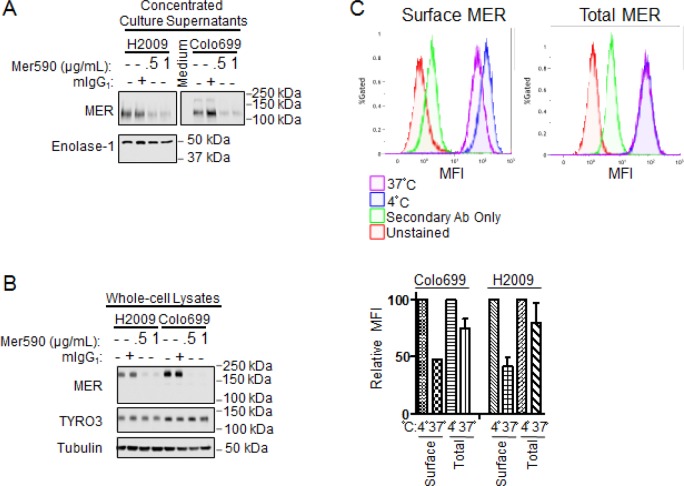
Mer590 induces internalization of surface MER (A and B) Colo699 and H2009 cells were cultured overnight in RPMI containing 10% FBS. The next morning, the medium was replaced with serum-free RPMI containing vehicle control (PBS), isotype antibody control (1 μg/ml mIgG_1_), or Mer590 (0.5 or 1 μg/ml). After 24 hours, the culture supernatants were collected, filtered to remove any floating cells and cellular debris, and concentrated approximately 40-fold using Amicon Ultra-4 centrifugal filter units (Millipore UFC803096). Adherent cells were lysed separately. MER protein levels were analyzed by western blot. (A) MER protein levels in concentrated culture supernatants. Enolase-1 is excreted from NSCLC cells and was used as a loading control [31]. (B) MER protein levels in whole-cell lysates demonstrate significant loss of total cellular MER following Mer590 treatment without affect on expression of the related receptor tyrosine kinase, TYRO3. Tubulin was used as a loading control. (C) Colo699 and H2009 cells were treated with Mer590 (2 μg/ml) for 20 minutes at 4°C to allow binding to MER without induction of internalization. Cells were then either kept at 4°C or shifted to 37°C for 20 minutes to allow for internalization before fixing in paraformaldehyde. Half of the samples were then stained for surface MER, while half were permeabilized and stained for total MER, and then analyzed by flow cytometry. Representative histograms are shown above, with quantification of median fluorescence index (MFI) values below. MFI values relative to samples kept at 4°C were determined, such that loss of MER in each compartment upon shifting to 37°C could be assessed.

Possible explanations for the reduction of total MER protein levels in response to Mer590 treatment include promotion of MER extracellular domain (ECD) shedding, and induction of MER internalization and degradation. A number of antibodies have been developed to target the MET receptor tyrosine kinase, and both mechanisms of action have been demonstrated, depending on the specific antibody utilized [28,29]. Additionally, the MER ECD can be cleaved from the cell surface under basal conditions, and ECD shedding is increased in response to stimulation with lipopolysaccharide or phorbol 12-myristate 13-acetate, posing the possibility that MER ECD shedding may also be induced by Mer590 administration [30]. To distinguish between these two mechanisms, levels of MER ECD in culture media with and without Mer590 were measured by western blot analysis (Figure 2A). Soluble MER protein was visualized at the expected molecular weight of 120-130 kDa [30]. Administration of Mer590 resulted in decreased MER ECD levels in the culture media compared to mIgG1 treated cells. Whole cell lysates were used to confirm efficacy of MER decrease by Mer590 (Figure 2B). The reduction of MER ECD in conditioned media would not be expected if the action of Mer590 was to increase receptor cleavage. The result is consistent with Mer590-induced receptor internalization, reducing surface MER available for cleavage.

To further test the hypothesis that Mer590 induces receptor internalization, cells were incubated with Mer590 at 4° C for 20 minutes. Although antibody binding occurs at this temperature, receptor internalization cannot [32]. Half of the samples were subsequently kept at 4° C and half were moved to 37° C for 20 minutes, a temperature permissible for receptor internalization. Cells were then fixed and either stained to detect surface MER, or permeabilized and stained to detect total MER. Median fluorescence intensity (MFI) levels were then determined by flow cytometry. After moving to the permissive temperature, cell surface levels of MER decreased by 52.6% and 58.4%, in the Colo699 and H2009 cell lines, respectively, while total MER levels were decreased by only 24.9% and 20.0% (Figure 2C) If Mer590 induced receptor cleavage, an equal loss would be expected from the cell surface and total levels; however, the selective loss of MER from the cell surface is consistent with receptor internalization. The small loss of total MER levels may be due to lysosomal degradation, the end-point of receptor internalization, taking place within the 20 minutes at the permissive temperature prior to fixation.

Together, the decreased MER ECD shedding into the media, and the decreased surface:total MER ratio, demonstrate that Mer590 promotes receptor internalization.

### Mer590 Prevents MER Phosphorylation and Downstream Signaling

**Figure 3:**
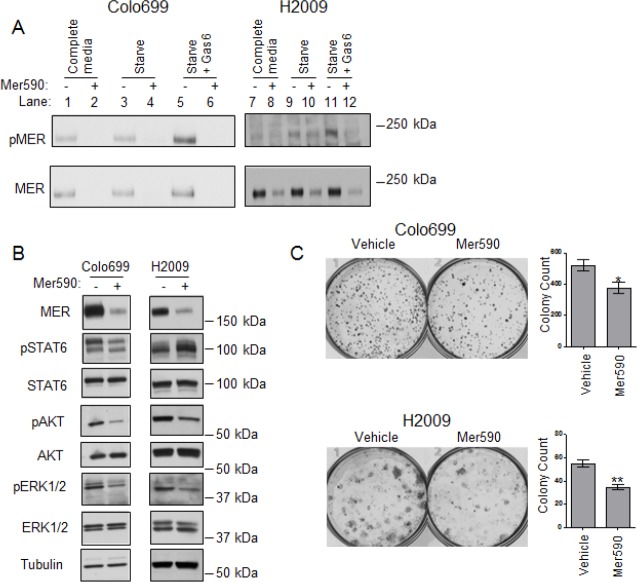
Mer590 inhibits ligand-dependent phosphorylation, activation of downstream signaling pathways, and colony formation in NSCLC cells (A) Colo699 and H2009 cells were cultured in the absence (−) or presence (+) of 0.5 μg/ml Mer590 for 48 hours followed by 2 hours in growth media containing 10% (complete media, lanes 1,2,7, 8) or 0% fetal bovine serum (serum starve, lanes 3-6, 9-12) and with or without Mer590. Samples in lanes 5, 6, 11, and 12 were then stimulated with 200 nM rhGas6 for 10 minutes. All cultures were treated with 120 μM pervanadate prior to cell lysis in order to stabilize MER phosphorylation. MER was immunoprecipitated from lysates and samples were analyzed by western blot using phospho-specific and total MER antibodies. (B) Colo699 and H2009 cells were cultured for 24 hours in serum-free medium containing Mer590 (2 μg/ml) or PBS vehicle. 200 nM rmGas6 was added for 10 minutes and cell lysates were prepared. Phospho-STAT6, phospho-AKT, and phospho-ERK1/2 levels were determined by western blot. Blots were stripped and re-probed for total protein levels. (C) Colo699 and H2009 cells were treated with Mer590 (2 μg/ml) or vehicle control for 72 hours, then lifted, stained with trypan blue, and counted. One thousand live cells were re-plated in complete media without Mer590 and cultured. Colonies were stained with crystal violet and counted after 10 days. Mean and SEM from at least three independent experiments are shown in the histograms to the right (*P<0.05, **P<0.01).

MER is activated by several ligands, including Gas6, which induces receptor autophosphorylation and activation of a wide variety of downstream signaling pathways [22]. To determine if Mer590 interferes with these signaling processes, cells were pre-treated with Mer590 or vehicle control for 48 hours, then cultured with or without serum in the continued absence or presence of Mer590 for two hours. At this point, cells were stimulated with either Gas6 or vehicle control for ten minutes. Cells were lysed and total and phospho-MER levels assessed by immunoprecipitation and western blotting (Figure 3A). As expected, Gas6 induced MER phosphorylation; this was prevented by Mer590 treatment (lanes 5,6, 11, and 12). Mer590 also reduced basal levels of phospho-MER in H2009 cells cultured in complete medium (lanes 7,8), as well as residual levels of phospho-MER in serum starved H2009 cells (lanes 9,10). In Colo699 cells, both phospho-MER and total MER were undetectable in samples treated with Mer590 (lanes 2, 4, and 6). To determine if inhibition of MER activation translated to reduced downstream signaling, cells were treated with Mer590 for 24 hours in the absence of serum, then stimulated with Gas6 for ten minutes. Phosphorylated and total STAT6, AKT, and ERK1/2 levels were then assessed by western blot. Mer590 pre-treatment resulted in decreased levels of Gas6-activated STAT6, AKT, and ERK1/2 in the Colo699 cell line, and decreased levels of Gas6-activated AKT and ERK1/2 in the H2009 cell line (Figure 3B).

### NSCLC Colony Formation is Reduced by Mer590 Treatment

To determine the long-term effects of Mer590, we utilized a re-plating assay. In this experiment, cells were treated for 72 hours with Mer590 or vehicle control, and then counted. Equal numbers of live cells were then re-plated at low density in fresh media and allowed to grow and form colonies for 10 days in the absence of any treatment. This experiment determines the residual effects of Mer590 treatment on cells that have survived the initial treatment period, but may be compromised in their ability to re-populate when Mer590 is withdrawn. As demonstrated in Figure 3C, treatment with Mer590 significantly decreased Colo699 colony number by 27.8% (p=0.0353). These results were confirmed in a second cell line, H2009, in which administration of Mer590 reduced colony formation in the re-plating assay by 36.8% (p=0.0013).

### Mer590 Enhances Carboplatin-Induced Apoptosis

**Figure 4:**
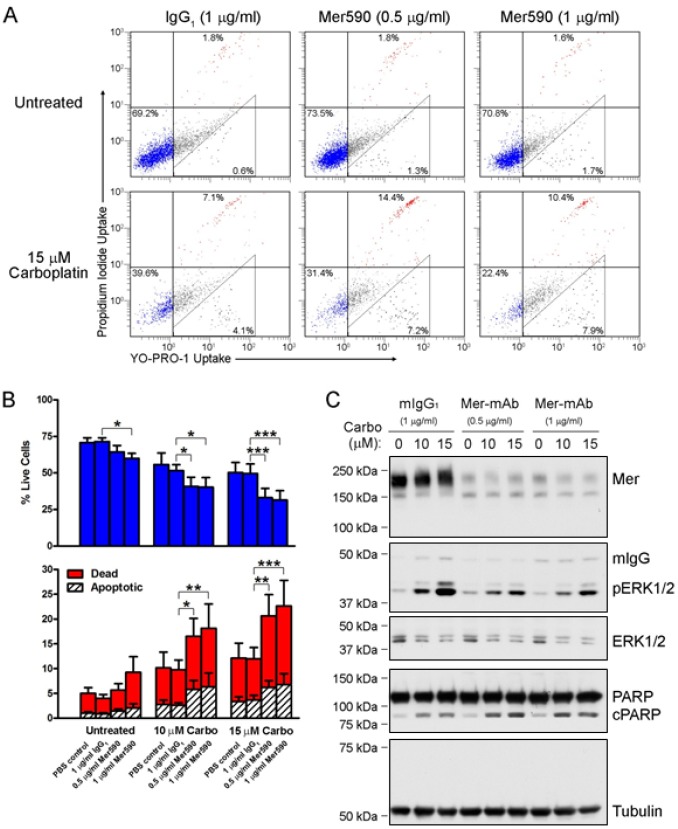
Mer590 increases carboplatin-induced apoptosis and decreases compensatory downstream pro-survival signaling Colo699 cells were cultured in the presence of vehicle (PBS), isotype antibody control (mIgG_1_), or Mer590 at the indicated concentrations for 48 hours followed by an additional 72 hours of treatment with antibody ± carboplatin. Apoptotic and dead cells were identified by flow cytometric analysis of YO-PRO-1 and PI uptake. (A) Representative histograms are shown. Early apoptotic cells are stained with YO-PRO-1 but are impermeable to PI. Dead cells and cells in late apoptosis are permeable to both dyes. Viable cells are not stained by either dye. The percentages of live (lower left quadrant), apoptotic (triangular gate), and dead (both upper quadrants) are shown. (B) Mean values and standard errors from 7 independent experiments are shown. Results were evaluated for significance using 2-way repeated measures ANOVA and Bonferroni posttests (*P<0.05, **P<0.01, ***P<0.001). No significant differences between PBS and mIgG_1_ controls were observed. (C) Whole cell lysates were prepared and expression of the indicated proteins was determined by western blot analysis. Blots representative of 3 independent experiments are shown.

Carboplatin and cisplatin are commonly administered with pemetrexed as the standard of care regimen for patients with NSCLC [33]. We determined whether Mer590 increased induction of apoptosis in NSCLC cells in response to treatment with chemotherapy. Colo699 cells were treated with vehicle control, mIgG_1_, or Mer590 in the presence or absence of 10 μM or 15 μM carboplatin. Live, early apoptotic, and dead cells were quantified by flow cytometry after staining with YoPro-1-iodide and propidium iodide (Figure 4A,B). Treatment with 1μg/ml Mer590 alone reduced the number of live cells from 71.5% to 60.0% compared to mIgG_1_ treated cells (p=0.0214), while 10 μM or 15 μM carboplatin (plus mIgG_1_) reduced the percentage of live cells to 51.6% and 49.6%, respectively (p=0.0015, p=0.0091). Treatment with a combination of Mer590 and 10 μM or 15 μM carboplatin resulted in the greatest induction of cell death, with only 40.3% and 31.5% live cells remaining, respectively (p=0.0009, p<0.0001). Induction of apoptotic cell death was confirmed biochemically by western blot analysis of PARP cleavage, which was highest in cells treated with both carboplatin and Mer590. (Figure 4C). Additionally, carboplatin alone induced a dose-dependent increase in pERK levels. However, Mer590 administration reduced carboplatin-induced pERK activation, potentially blocking a pathway used to escape carboplatin-induced apoptosis.

### Dual MER Inhibition Synergizes with Carboplatin to Induce Cell Death

**Figure 5:**
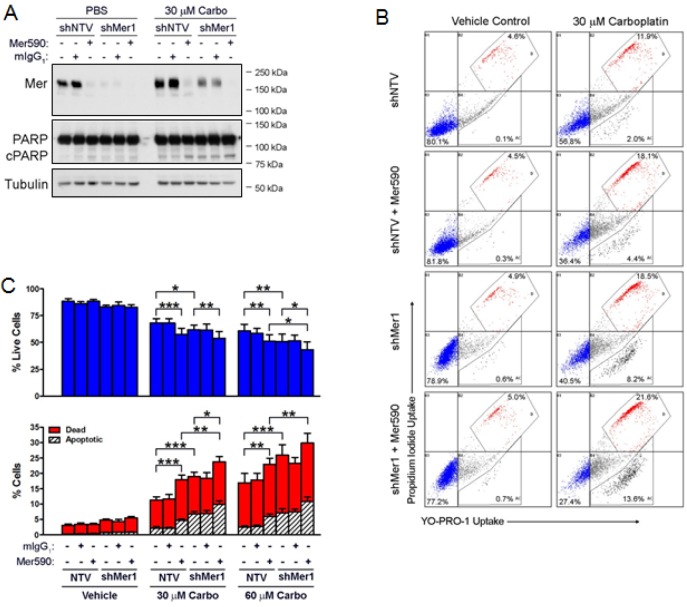
Mer590 has combinatorial effects with shRNA against MER to promote carboplatin-induced apoptotic cell death of NSCLC cells Colo699 cells expressing shRNA against MER (shMer1) or non-targeting control vector (shNTV) were pre-treated with 0.5 μg/ml Mer590 or mIgG_1_ for 48 hours, followed by an additional 72 hours of treatment with antibody plus 30 μM carboplatin or vehicle control (PBS). (A) Whole cell lysates were prepared and expression of the indicated proteins (cPARP = cleaved PARP) was determined by western blot analysis. Blots representative of 3 independent experiments are shown. (B) Apoptotic and dead cells were identified by flow cytometric analysis of YO-PRO-1 and PI uptake. Representative histograms are shown. The percentages of apoptotic and dead cells are derived from the lower and upper polygonal gates, respectively. (C) Mean values and standard errors from 6 independent experiments are shown. Results were evaluated for significance using 2-way repeated measures ANOVA and Bonferroni posttests (*P<0.05, **P<0.01, ***P<0.001). No significant differences between PBS control and mIgG_1_ control were observed (data not shown).

As Mer590 increased induction of apoptosis in response to carboplatin, we hypothesized that greater MER inhibition would lead to an even more substantial increase in apoptosis induced by carboplatin. shRNA was used to stably knock down MER in the Colo699 cell line (Figure 5A). Both shRNA and Mer590 alone induced down-regulation of total MER. However, when administered together, shRNA and Mer590 reduced total MER further (Figure 5A). shRNA against MER combined additively with both 30 μM and 60 μM carboplatin (p=0.056 and p=0.055, vs. additivity), while Mer590 combined synergistically with 30 μM carboplatin (p=0.002) and additively with 60 μM carboplatin (p=0.079) (Figure 5B,C and Table 1). The combination of both shRNA and Mer590, in a dual-MER inhibition strategy, synergized with both 30 μM and 60 μM carboplatin (p=0.030 and p=0.009) (Figure 5B,C and Table 1). This finding was confirmed via western blot analysis of PARP cleavage, which was highest when dual MER inhibition was combined with carboplatin (Figure 5A). Interestingly, carboplatin treatment increased total levels of MER expression (Figure 5A), possibly as a compensatory survival response. The chemotherapy-induced MER up-regulation may reflect an increased reliance on MER signaling in the presence of carboplatin, and provide a rationale for carboplatin and MER-targeted agent combinations.

**Table 1:** Dual MER inhibition (Mer590 plus shRNA) interacts synergistically with carboplatin to induce apoptosis and cell death in NSCLC cells The percentages of apoptotic and dead cells are derived from the polygonal gates labeled AC and D, respectively, in Figure 5. For the purpose of this comparison, the sum of the apoptotic and dead percentages were used (%ApD = red bars + hatched bars from Fig. 5). Baseline cell death observed in untreated samples was subtracted from raw %ApD. Synergism between carboplatin and a single MER inhibitor (Mer590 or shMer) was evaluated independently from synergism between carboplatin and dualMER inhibition (Mer590 + shMer). The expected %ApD for an additive interaction was determined using the Bliss additivity model and is shown (Additive) [34]. Statistically significant increases (student's paired t test p value < 0.05) in the %ApD observed after MER inhibition in combination with carboplatin relative to the expected additive %ApD indicate synergy. Mean values and standard errors were derived from 4 independent experiments

Mer590	shMER	Dual MER	30 μM Carboplatin	60 μM Carboplatin	Additive	Combination	P Value
0.18 ± 0.2	-	-	8.56 ± 1.4	-	8.72 ± 1.5	15.36 ± 1.4	0.002
-	1.76 ± 0.3	-	8.56 ± 1.4	-	10.16 ± 1.5	15.94 ± 2.2	0.056
-	-	2.45 ± 0.6	8.56 ± 1.4	-	10.79 ± 1.5	20.27 ± 2.9	0.030
0.18 ± 0.2	-	-	-	13.65 ± 3.2	13.79 ± 3.3	19.71 ± 2.4	0.079
-	1.76 ± 0.3	-	-	13.65 ± 3.2	15.18 ± 3.1	22.61 ± 4.4	0.055
-	-	2.45 ± 0.6	-	13.65 ± 3.2	15.79 ± 3.0	26.56 ± 4.3	0.009

## DISCUSSION

Our group has previously validated MER as a potential therapeutic target in NSCLC by demonstrating that shRNA-mediated MER knockdown results in decreased pro-oncogenic signaling, synergy with standard chemotherapeutic agents, increased induction of apoptosis, and decreased colony and tumor formation in long-term assays [13]. In this study, we furthered this work by demonstrating that a clinically relevant therapeutic agent, a novel anti-MER monoclonal antibody, can phenocopy the effects of genetic inhibition of MER.

Mer590 treatment of NSCLC cells resulted in reduced surface and total levels of MER. This effect occurred rapidly (over one to four hours), lasted for up to seven days, and could be achieved with concentrations in the low nanogram/milliliter range. MER inhibition translated to a decrease in phosphorylated MER and a concurrent decrease in activation of downstream pro-oncogenic signaling molecules, including STAT6, AKT, and ERK1/2. Functional effects of these biochemical changes included increased apoptosis and decreased colony formation, mediated by Mer590 as a single agent and in combination with carboplatin or shRNA against MER.

The apoptosis and re-plating assays used here complement each other and highlight the ability of Mer590 to induce both short-term increases in apoptosis and the delayed effect of decreased colony forming potential even in cells that survive the initial treatment period. This residual defect in colony formation could theoretically be important in reducing the ability of cancer cells to repopulate leading to recurrence after a treatment cycle.

An additional feature of clinically relevant MER-targeted therapy is a potential increased efficacy of standard chemotherapeutics, which have previously been shown to interact synergistically with shRNA-mediated MER inhibition in NSCLC, and with small molecule-mediated MER inhibition in ALL [13, 25]. In this study, Mer590 interacted synergistically with carboplatin, a commonly used chemotherapy for NSCLC treatment. These data indicate the possibility that co-administration of MER-targeted agents with carboplatin may decrease tumor burden more effectively than carboplatin alone. Alternatively, utilization of MER-targeted agents may allow for dose-reduction of standard chemotherapy, with the goal of fewer or less severe side effects for NSCLC patients.

In addition to chemotherapy, Mer590 could also be administered in combination with alternative mechanisms of MER inhibition. Mer590 plus shRNA against MER synergized with carboplatin to cause increased apoptosis. A dual inhibition strategy against a single receptor has been successfully utilized against the ERBB family of receptors, where the antibodies cetuximab, panitumumab, or trastuzumab have been combined with small molecule inhibitors, including gefitinib, erlotinib, afatinib, or lapatinib, in several clinical trials [35]. The combination of cetuximab and afatinib showed promising results in a phase Ib/II trial in NSCLC, and cetuximab paired with either gefitinib or erlotinib has generated promising data in patients with colon cancer. The most impressive clinical data thus far; however, have been generated in breast cancer, in which the combination of trastuzumab plus lapatinib has increased survival in two phase III clinical trials. In one trial, the effect was compared to lapatinib alone, while in the second, where the drug combination was given in combination with paclitaxel, survival was increased as compared to paclitaxel plus either targeted therapy alone.

In conclusion, we have developed a novel monoclonal antibody, Mer590, that inhibits MER receptor tyrosine kinase, a recently validated target in NSCLC. This manuscript describes critical pre-clinical experiments demonstrating the mechanism of action and the therapeutic potential of this antibody. Treatment with Mer590 is sufficient to induce apoptotic cell death and reduce colony formation and these effects can be augmented when given in conjunction with carboplatin or genetic inhibition of MER. Taken together, these data validate antibody-mediated targeting of MER as an attractive strategy for treatment of lung cancer that deserves further optimization and investigation.

## MATERIALS AND METHODS

### Cell culture and treatment

All cell lines were cultured in RPMI medium supplemented with 10% fetal bovine serum (FBS), penicillin (100 U/ml) and streptomycin (100 μg/ml). Recombinant human (rhGas6, 885-GS) and mouse (rmGas6, 986-GS) Gas6 were purchased from R&D Systems. Carboplatin was purchased from Sigma (C2538). NSCLC cell lines were purchased from the American Type Culture Collection (ATCC, Manassas, VA: A549 and H2009) or from the German Collection of Microorganisms and Cell Cultures (DSMZ, Braunschweig, Germany: Colo699 and HCC15). All parental cell lines and shRNA-transduced derivatives (polyclonal and clonal) used in these studies were subjected to short tandem repeat (STR) analysis and the profiles were compared to publically available databases to verify their authenticity.

### Western blotting

Adherent cells were either lifted with 0.02% EDTA in PBS and resuspended in lysis buffer (50 mM HEPES pH 7.5, 150 mM NaCl, 10 mM EDTA, 10% glycerol, 1% Triton X-100, 1 mM Na_3_VO_4_) supplemented with protease inhibitors (Complete Mini, Roche Molecular Biochemicals), or washed with PBS and scraped into lysis buffer. Total protein concentrations were determined and western blotting was performed as previously described [13].

### Antibodies for western blotting and flow cytometry

For western blotting, the following antibodies were obtained from Cell Signaling: pAKT (S473, Cat# 9271), AKT (Cat# 9272), Enolase-1 (Cat# 3810), pERK1/2 (T202/Y204, Cat# 9106), ERK1/2 (Cat# 9102), PARP (Cat# 9542), pSTAT6 (Y641, Cat# 9361), STAT6 (Cat# 9362), TYRO3 (Cat# 5585), and α-tubulin (Cat# 2125). Additional antibodies used for western blotting include AXL (R&D Systems, AF154), pMER (Y749, Y753, Y754, PhosphoSolutions), and MER (Abcam 52968). Horseradish peroxidase (HRP)-conjugated secondary antibodies (goat anti-rabbit, Bio-Rad 170-6515; goat anti-mouse, Bio-Rad 170-6516; donkey anti-goat, Santa Cruz sc-2020) were used for enhanced chemiluminescence of western blots. The following antibodies were used for measurement of indirect immunofluorescence using flow cytometry: mouse monoclonal anti-MER (Caveo Therapeutics, CVO-590) and phycoerythrin-conjugated donkey anti-mouse (Jackson Immunoresearch, 715-116-150) (Figure 1) or Mer590 and allophycocyanin-conjugated donkey anti-mouse (Jackson Immunoresearch, 715-136-150) (Figure 2). All antibodies were used as recommended by the manufacturer unless otherwise specified.

### Immunoprecipitation and detection of phosphorylated MER

Cells were treated with the phosphatase inhibitor pervanadate (0.12mM Na_3_VO_4_ in 0.002% H_2_O_2_ in PBS) for 5 minutes (H2009) or 1 minute (Colo699), and then lysed. Lysates were incubated with antibodies against MER (R&D Systems MAB8912) and rec-Protein G-sepharose 4B beads (Invitrogen 10-1242) overnight. Beads were washed twice with lysis buffer, and bound proteins were eluted by boiling in Laemmli buffer (62.5 mM Tris-HCl pH 6.8, 25% glycerol, 5% beta-mercaptoethanol, 2% SDS, and 0.01% bromophenol blue). Proteins were resolved on SDS-polyacrylamide gels and phosphorylated MER protein was detected by western blot. Blots were stripped and re-probed to determine total MER protein levels (Abcam 52968).

### Assessment of downstream signaling

Sub-confluent cultures (approximately 5 × 10^5^ cells) were pre-treated with 2 μg/mL Mer590 or PBS vehicle control in serum-free RPMI culture medium for 24 hours. 200 nM rmGas6 was then added to each well for 10 minutes before cells were lysed. Lysates were quantified by Bradford assay and analyzed by western blot.

### Flow cytometric detection of surface and total proteins

Sub-confluent cultures (approximately 5 × 10^5^ cells) were washed with PBS and lifted with 0.02% EDTA in PBS. Harvested cells were fixed in 4% paraformaldehyde (Figure 2C) or not (Figure 1), then washed in FACS wash buffer (2% FBS and 0.02% azide in PBS) prior to staining in 50 μl staining solution (1% FBS and 0.02% azide in PBS) containing primary antibody, murine immunoglobulin (mIgG_1_, R&D Systems, MAB002), or vehicle control, for 15-30 minutes at 4°C. Cells were washed again in FACS wash buffer, and then incubated in staining solution containing fluorophore-conjugated secondary antibody or vehicle control for 15-30 minutes at 4°C. Stained cells were washed in FACS wash buffer, resuspended in staining solution, and fluorescence of surface-bound antibodies was measured by flow cytometry. For assessment of total MER, cells were fixed in 4% paraformaldehyde, washed in FACS wash buffer, permeabilized in permeabilization/wash buffer (BD, 554723), resuspended in staining solution containing secondary antibody or vehicle control for 30 minutes at 4°C, washed in perm/wash buffer, and resuspended in staining solution before analysis by flow cytometry.

### Lentiviral transduction and isolation of clonal populations

Lentiviral vectors (pLKO.1) containing shRNA sequences targeting MER (shMer1, Oligo ID: TRCN0000000862), or non-silencing control GFP (shControl, catalog no. RHS4459) were obtained from Open Biosystems. Lentiviral particles were produced in 293FT cells and Colo699 target cells were transduced as previously described [13]. Polyclonal populations were maintained in selection medium containing puromycin (2 μg/ml). Stable clonal isolates were obtained by single-cell sorting using flow cytometry. Clonal populations were cultured in puromycin for 2-3 doubling times every 2-3 weeks.

### Apoptosis and cell death assay

Sub-confluent cultures were treated with mIgG_1_or Mer590 for 48 hours, then mIgG_1_ or Mer590 with or without carboplatin for an additional 72 hours. Supernatants were collected and combined with cells after lifting with EDTA. Cells were then stained with 0.2 uM YO-PRO-1 and 1.5 uM propidium iodide (PI) (Invitrogen). Uptake of dyes was assessed by flow cytometry using an FC500 flow cytometer and CXP analysis software (Beckman Coulter).

### Re-plating colony formation assay

Sub-confluent cultures were treated with vehicle control or Mer590 for 72 hours. Cells were then lifted with EDTA and counted. One thousand viable cells, as determined by trypan blue exclusion, were cultured in 6-well plates. Colonies were stained with crystal violet and counted after 10 days.

### Production of monoclonal antibody

The anti-MER monoclonal antibody (Mer590) was purified from a mouse hybridoma generated by fusion of FoxNY mouse myeloma cells to B-cells from Balb/C mice immunized with recombinant MER extracellular domain/Fc chimera as previously described [27].

### Statistical Analysis

Statistical analysis was performed using Prism 5 software (GraphPad Software, Inc.). All data are representative of at least three independent experiments.
